# Universal Properties and Specificities of the β_2_-Adrenergic Receptor-G_s_ Protein Complex Activation Mechanism Revealed by All-Atom Molecular Dynamics Simulations

**DOI:** 10.3390/ijms221910423

**Published:** 2021-09-27

**Authors:** Argha Mitra, Arijit Sarkar, Attila Borics

**Affiliations:** 1 Laboratory of Chemical Biology, Institute of Biochemistry, Biological Research Centre, 62. Temesvári krt., H-6726 Szeged, Hungary; argha.mitra@brc.hu (A.M.); sarkar.arajit@brc.hu (A.S.); 2 Theoretical Medicine Doctoral School, Faculty of Medicine, University of Szeged, 97. Tisza L. krt., H-6722 Szeged, Hungary

**Keywords:** GPCR, adrenergic, activation mechanism, signal transduction, molecular dynamics

## Abstract

G protein-coupled receptors (GPCRs) are transmembrane proteins of high pharmacological relevance. It has been proposed that their activity is linked to structurally distinct, dynamically interconverting functional states and the process of activation relies on an interconnecting network of conformational switches in the transmembrane domain. However, it is yet to be uncovered how ligands with different extents of functional effect exert their actions. According to our recent hypothesis, based on indirect observations and the literature data, the transmission of the external stimulus to the intracellular surface is accompanied by the shift of macroscopic polarization in the transmembrane domain, furnished by concerted movements of highly conserved polar motifs and the rearrangement of polar species. In this follow-up study, we have examined the β_2_-adrenergic receptor (β_2_AR) to see if our hypothesis drawn from an extensive study of the μ-opioid receptor (MOP) is fundamental and directly transferable to other class A GPCRs. We have found that there are some general similarities between the two receptors, in agreement with previous studies, and there are some receptor-specific differences that could be associated with different signaling pathways.

## 1. Introduction

G protein-coupled transmembrane receptors (GPCRs) constitute one of the largest and most important protein superfamilies of the human genome. Their importance mainly derives from their remarkably high pharmacological relevance [1,2,3]. GPCRs are classified into six sub-families (A-F) based on their sequence and function. From the structural perspective GPCRs share similar architecture in their transmembrane domains and possess high sequential and structural diversity of their extra- and intracellular loops and the extracellular (*N*-terminal) and cytosolic (*C*-terminal) domains. These variable domains are proposed to be responsible for ligand and G protein/arrestin specificity, whereas the transmembrane (TM) domain controls the transmission of external signals to the intracellular surface of the protein. The variety of G proteins that mediate GPCR signaling is very low relative to the diversity of GPCRs and their external activators. Therefore, the activation of GPCRs is suggested to follow a general structural mechanism.

Adrenergic receptors, responsible for the homeostasis between stressful and resting conditions of the body, belong to the most populated class (A) of GPCRs. The β_2_-adrenergic receptor (β_2_AR) is linked to respiratory, as well as other smooth muscle relaxation. These receptors are stimulated by endogenous neurotransmitters, such as epinephrine and norepinephrine and the signal transduction of β_2_AR is mediated predominantly by the G_s_ (stimulatory) protein complex [4]. The agonist-β2AR-Gs signaling triad is an important target of drug development activities to treat severe respiratory conditions, such as chronic obstructive pulmonary disease (COPD) or asthma. Remarkable efforts have been invested in the optimization of GPCR targeting drugs to reduce their undesired side effects. Such efforts necessitated the in-depth structural analysis of target GPCR structures [2].

The β2AR was among the first GPCRs of which a three-dimensional structure was solved at atomic resolution [5] and numerous experimental studies have been conducted to elucidate the structural details of activation of this TM receptor [6,7,8,9,10,11,12,13,14,15]. To date, the β2AR is the most widely investigated class A GPCR and is frequently used as a universal model for the study of the structural mechanism of activation for class A GPCRs [16]. Recent developments in the field of experimental structural biology led to the rapid accumulation of experimental data of GPCR structures which are now readily available in the Brookhaven Protein Data Bank as well as in GPCRdb, a specific, comprehensive collection of all GPCR structures published to date (http://gpcrdb.org, accessed on 23 August 2021) [15]. A general structural difference between active and inactive state class A GPCRs, including β2AR, is the position of the sixth helix of the TM domain (TM6) [5,7,8]. The capabilities of conventional experimental techniques to provide information about the mechanism of transition between these structural states are limited. The current theory of the structural mechanism of class A GPCR activation was framed on the basis of results acquired through the application of state-of-the-art molecular dynamics (MD) simulation hardware and techniques [17,18,19]. According to this theory, GPCRs exist in a dynamic ensemble of multiple active, inactive, and intermediate states, even in the absence of ligands. The populations of these states are shifted upon ligand binding, depending on the functional properties of the bound ligand. The fifth, sixth, and seventh TM helices (TM5, TM6, TM7, respectively) have been emphasized for their foremost interplay in signal transduction [16,20].

As well as the rearrangement of TM helices, highly conserved polar functional motifs, E/DRY, NPxxY, and CWxP, have been appointed as participants of the activation mechanism [21,22]. Specific rearrangements of intramolecular interactions involving these motifs, the conserved allosteric Na+ binding site [23,24], and the extended network of water molecules in the internal cavities connecting the orthosteric ligand-binding pocket to the cytosolic domains have been proposed recently as general machinery of signal transmission in GPCRs [25,26]. In order to respond to the most recent challenge of rational drug design and to develop high-affinity, high-efficacy, and functionally selective GPCR ligands a quantitative model of the activation mechanism is necessary. Models built exclusively on a structural basis have limited capabilities to differentiate between ligands with similar structure, physico-chemical properties, and binding affinity, but different efficacy. Consequently, the creation of a directly transferable model would need the introduction of new perspectives.

Our recent results of extensive MD simulations of the μ-opioid receptor (MOP) indicated that the dynamic motions of polar amino acid side chains of conserved motifs are highly correlated [27]. Such concerted motions were only observed during the simulations of the agonist- and Gi protein-bound active state MOP, suggesting that this phenomenon could be associated with the signal transduction process, corroborating the above-cited proposal [25,26]. These polar amino acid side chains of the orthosteric and allosteric binding pockets, the NPxxY and E/DRY motifs and the cytosolic helix (H8), form a polar signaling channel connecting the binding pocket to the intracellular G protein-binding surface. Frequent transitions between rotameric states, however, were not observed for these specific side chains, which casts doubt on the channel’s operation as a sequential conformational switch. According to our recent proposal, receptor activation is accompanied by a shift of macroscopic polarization in a shielded central duct of the TM domain, initiated by ligand binding and propagated by the minuscule rearrangements of polar amino acid side chains along TM7. TM7 was further implied in the activation mechanism as a potential conductor owing to its inherent dipole moment. Evidently, MD simulations employing fixed point charge force fields cannot reveal exact or quantitative details of processes involving charge shift. Nevertheless, independent mutation data provided convincing support for the interplay of the above-mentioned polar species [27].

Here we present an extensive, unbiased, atomistic MD simulation study of the full sequence β2AR, embedded in a native-like caveolar membrane bilayer, in the presence of an endogenous agonist and the Gs protein complex or β-arrestin-2. Simulations were started from the active and inactive structural form of the receptor, revealed by previous X-ray crystallographic studies [5,7]. Analyses were conducted according to the above-mentioned novel perspective, to examine that our previously proposed indirect hypothesis could be extended to other class A GPCRs.

## 2. Results and Discussion

### 2.1. Simulation System Integrity

On the grounds that the disposition of TM helices was proposed to have a pivotal role in the activation mechanism [16,20,25,26], an important specific aim of this current study was to study the full sequence β2AR in order to take account of the pull of the *N*- and *C*-terminal domains posed on the TM helices and to see if that affects the internal dynamics of the TM domain. These highly variable and flexible domains are generally omitted in the experimental structures of GPCRs and, consequently, from the corresponding MD simulation studies. Here, approximate structures of the terminal domains were generated through folding simulations (see Section 3). Even if parallel folding simulations provide convergent results, the correct and complete folding of these domains in the available time frame could not be guaranteed. Nevertheless, their effect on TM dynamics, primarily exerted by their mass is satisfactorily taken into account by using these approximate structures.

Unfolding of *N*- and *C*-terminal domains during simulations could result in contacts formed between the neighboring periodic images of these unfolded domains which could lead to artifacts. The evolution of the radii of gyration of *N*- and *C*-terminal domains indicated partial unfolding during some of the production simulations (Figure S1), but the minimum distance between the *N*- and *C*-terminal domains, was never below 1.4 nm (Figure S2). Therefore, the possibility of artificial contact between these domains could be excluded.

With regard to the stability of simulation systems, no dissociation or notable relative displacement of macromolecular components were observed during the simulations. Epinephrine, however, dissociated from the orthosteric binding pocket on two occasions. First, it was ejected from the binding pocket during the first 100 ns of one of the three replica simulations of the active Gs protein-bound β2AR. The second time it was observed for the inactive Gs protein-bound β2AR, when epinephrine left the orthosteric binding site after approximately 600 ns of simulation time.

**Figure 1:**
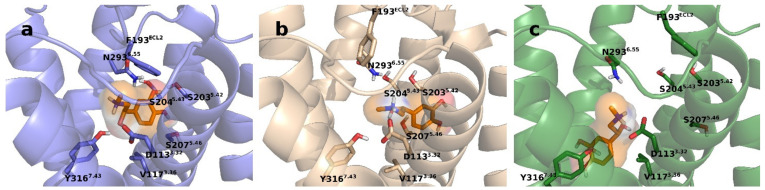
Ligand position in the orthosteric binding pocket in the 1st (**b**) and 2nd (**c**) replica simulations of the active β_2_AR–G_s_ protein–epinephrine complex in comparison with the crystallographic structure ((**a**), PDB code: 4LDO).

In the second replica simulation of the active G_s_ protein-bound β_2_AR, the ligand took an opposite orientation in the binding pocket, compared to the X-ray crystallographic structure [8] of the β_2_AR-epinephrine complex (Figure 1). These simulations were not excluded from analysis but the results were interpreted accordingly. A reference simulation of the active G protein-bound β_2_AR in which epinephrine was restrained to the binding pocket was performed in order to explain discrepancies emerging from ligand dissociation. The instability of β_2_AR–epinephrine complexes observed during some of the simulations may be explained by the smaller size and remarkably lower affinity of epinephrine relative to the ligands used in previous simulations of the β_2_AR (μM vs. pM range affinity, respectively) [17,28].

### 2.2. Allosteric Na^+^ Binding

**Figure 2:**
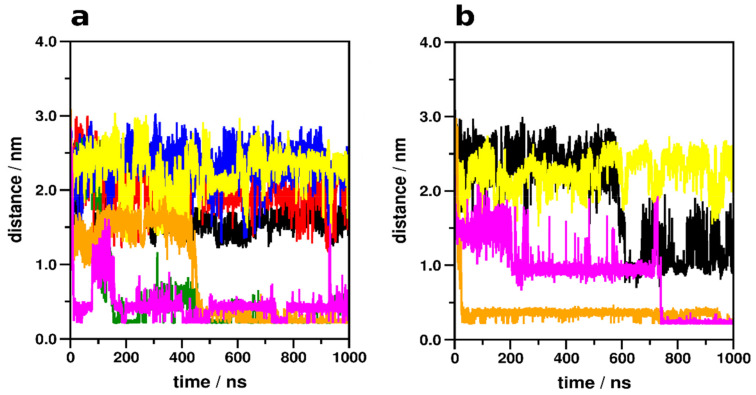
Minimum distance between Na^+^ ions and the allosteric Na^+^ binding site, D79^2.50^ of the active (**a**) and inactive state (**b**) β_2_AR during simulations. Black: β_2_AR–G_s_ protein–epinephrine complex, 1st replica; Red: β_2_AR–G_s_ protein–epinephrine complex, 2^nd^ replica; Green: β_2_AR–G_s_ protein–epinephrine complex, 3^rd^ replica; Blue: β_2_AR–G_s_ protein–epinephrine complex, with restrained epinephrine and GDP; Yellow: β_2_AR-β-arrestin-2–epinephrine complex; Orange: ligand-free β_2_AR–G_s_ protein complex; Magenta: ligand-free β_2_AR-β-arrestin-2 complex.

Na^+^ penetration to the allosteric Na^+^ binding site (D79^2.50^) (Figure 2, Table 1) was not observed for the epinephrine-bound β_2_AR, regardless of the state of the receptor, except when epinephrine dissociated from the orthosteric binding site during the course of the simulation. Furthermore, Na^+^ entrance from the cytosolic side did not occur in any of the systems. In the absence of epinephrine, however, localization of Na^+^ at the ortho- (D113^3.32^) (Figure S3) and allosteric sites took place. Frequent contacts were formed between Na^+^ and the ortho- and allosteric sites as well as residues of the conserved CWxP and NPxxY motifs, but no relevant trend of contact frequencies was identified which could be directly associated with the modulation of receptor activation by Na^+^ ions (Table 1). Interestingly, Na^+^ was occasionally present in the orthosteric binding pocket of the G_s_ protein- and epinephrine-bound, active state β_2_AR. This phenomenon may be less relevant since it was only noticeable in one of the simulation replicas (Figure S3) and the frequency of contact between Na^+^ and D113^3.32^ was negligible (Table 1). The presence of Na^+^ in the orthosteric pocket and the proximal CWxP motif was more prominent for the G_s_ protein- and epinephrine-bound inactive state β_2_AR. However, this was a clear consequence of ligand dissociation after approximately 600 ns of simulation time. No Na^+^ insertion to the ortho- or allosteric sites was observed in any of the ligand- and β-arrestin-2-bound states.

These observations are in complete agreement with previous MD simulation data of this receptor [29], and other class A GPCRs [27,30,31,32], and corroborate that the allosteric Na^+^ binding site is only accessible through the orthosteric binding pocket and the bound orthosteric ligand blocks the entrance of Na^+^ to the allosteric site. Intracellular access of Na^+^ ions through the TM domain is closed by the bound G_s_ protein complex or β-arrestin-2. Translocation of Na^+^ ions through the TM region was observed in previous MD simulations of the active state MOP in the absence of bound ligands and intracellular proteins, suggesting that it takes place during the process of receptor deactivation [33].

### 2.3. Transmembrane Helix and Loop Dynamics

In our previous study of the MOP, TM7 was found to be the most ordered among the TM helices of the active state G_i_ protein-bound receptor. Furthermore, the helical conformation of TM7 was indicated to be the closest to ideal when the receptor was bound to the G_i_ protein complex. Conversely, the lowest degree of order of TM7 was observed in the β-arrestin-2-bound state. Based on these geometric features it was assumed that TM7 possesses the highest dipole moment in the G_i_ protein-bound state, which could facilitate electron, proton, or ion conduction along the helix axis [34]. Such conduction capacity could be relevant for the proposed model, which involves the shift of charge balance between the orthosteric binding pocket and the intracellular G protein-binding interface during class A GPCR activation [27]. Such a proposed role of TM7 was not corroborated by the results obtained here for β_2_AR (Figure S4). As opposed to the MOP, TM7 was among the least ordered TM helices of the β_2_AR. Furthermore, the relatively high order of TM7 in the active, epinephrine- and G_s_ protein-bound state was not reproduced in the reference simulation of that system and was also matched by the β-arrestin-2 and epinephrine-bound receptor (Figure S4). This suggests that the above-described trend is a specific property of MOP and/or class A GPCRs signaling through the G_i_ protein complex.

Atomic displacement analysis of the TM6 of epinephrine-bound β_2_AR indicated moderate dispositions from the corresponding starting structures (Figures S5 and S6), similar to our previous simulation results for the MOP [27]. Remarkable rearrangements of TM helices have occurred during previous simulations of the β_2_AR, but at longer timescales and in the absence of bound intracellular proteins [17]. Slightly larger dispositions of TM6 were observed in the ligand-free systems, confirming the stabilizing effect of the bound agonist, again, in agreement with our previous results [27]. One exception was the β-arrestin-2-bound inactive receptor in which, similar to the ligand-bound systems, no significant TM6 disposition took place. This, in contrast with our previous results, suggests the preference of β-arrestin-2 for the inactive structure of this receptor.

**Figure 3:**
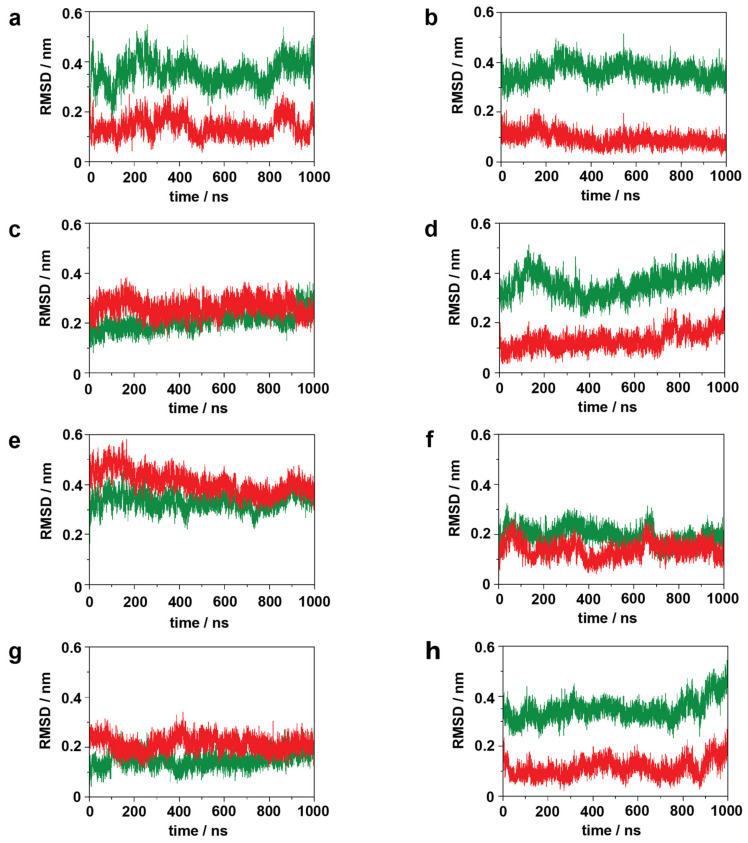
Disposition of the NPxxY motif during simulations with respect to the active (green) and inactive (red) crystallographic structures of the β_2_AR. (**a**) active β_2_AR–G_s_ protein–epinephrine complex, 1st replica; (**b**) inactive β_2_AR–G_s_ protein–epinephrine complex; (**c**) active β_2_AR–β-arrestin-2–epinephrine complex; (**d**) inactive β_2_AR–β-arrestin-2–epinephrine complex. (**e**) active, ligand-free β _2_AR–G_s_ protein complex; (**f**) inactive, ligand-free β_2_AR–G_s_ protein complex; (**g**) active, ligand-free β_2_AR–β-arrestin-2 complex; (**h**) inactive, ligand-free β_2_AR–β-arrestin-2 complex.

The unexpected disorder of TM7, discussed above, prompted us to analyze the disposition of this helical segment to see if the dynamics of the NPxxY motif show any correlation with the activation state or with the presence of agonist and/or intracellular signaling proteins. Surprisingly, large dispositions (~0.4 nm RMSD) of the NPxxY motif were found, relative to the active state structure and to the corresponding TM7 dispositions in several simulations (Figure 3, Figure S7).

**Figure 4:**
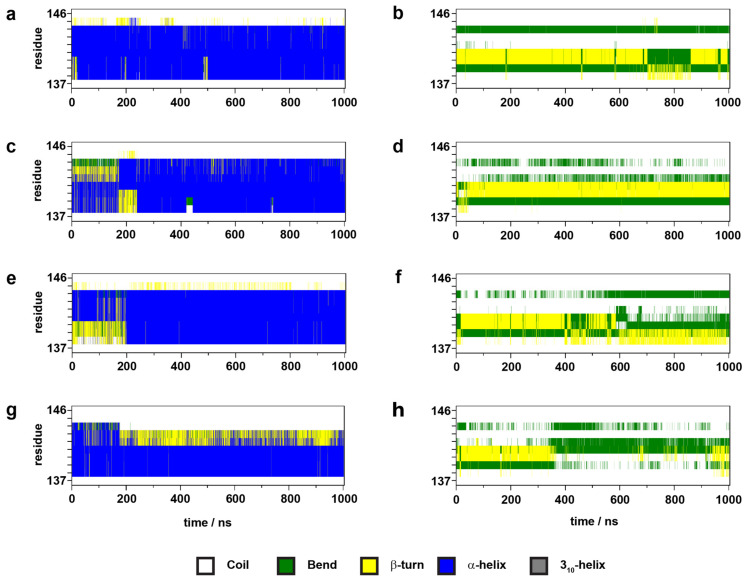
Evolution of the secondary structure of ICL2 during simulations. (**a**) active β_2_AR–G_s_ protein–epinephrine complex, 1st replica; (**b**) inactive β_2_AR–G_s_ protein – epinephrine complex; (**c**) active β_2_AR–β-arrestin-2–epinephrine complex; (**d**) inactive β_2_AR–β-arrestin-2–epinephrine complex. (**e**) active, ligand-free β_2_AR–G_s_ protein complex; (**f**) inactive, ligand-free β_2_AR–G_s_ protein complex; (**g**) active, ligand-free β_2_AR–β-arrestin-2 complex; (**h**) inactive, ligand-free β_2_AR–β-arrestin-2 complex.

Most interestingly, this large disposition of the NPxxY motif coincides with the intensive concerted dynamics of the second segment of the polar signaling channel, which could be associated either with receptor activation or constitutional activity (see data and discussion below). It should be noted, that the results of the epinephrine and G protein-bound inactive state receptor and the ligand-free G protein-bound inactive β_2_AR are very similar, due to epinephrine dissociation after 600 ns. The results of secondary structure analysis indicated that ICL1, ICL3, and H8 maintain their secondary structures in all receptor states and only minor, reversible changes occur, resulting from internal dynamics (Figures S8–S12). ICL2, on the other hand, adopted an α-helical structure in active states and got partially unfolded in inactive states (Figure 4, Figure S13). ICL2 of the active β_2_AR was recently shown to be α-helical when the receptor is bound by G_s_ and partially unfolded when the G_i_ protein complex is attached [35]. Similar signaling protein selectivity was observed previously for the MOP receptor [27]. However, in contrast with the present results, the structure of ICL2 did not demonstrate any dependence on the activation state of the MOP. Nevertheless, the present simulation results are in agreement with extensive experimental data reporting an α-helical structure of ICL2 in the active [7,8,36,37] and unfolded structure in the inactive state of β_2_AR [5,11].

### 2.4. Correlated Side-Chain Motions in the Transmembrane Domain

**Figure 5:**
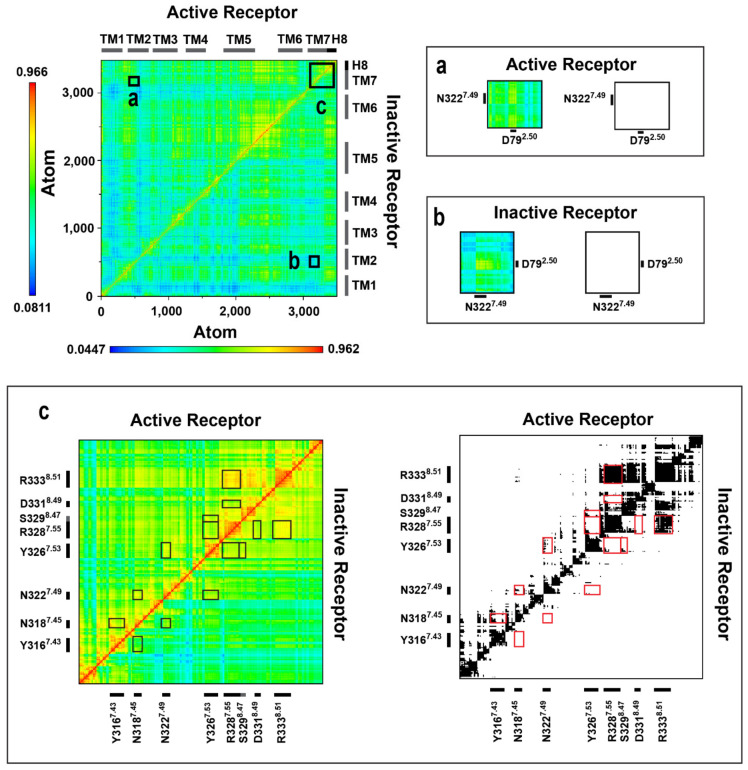
Dynamic cross-correlation matrices of the G_s_ protein-bound β_2_AR in active and inactive states. Panels (**a**–**c**) are magnified views of regions of amino acid residues of interest. Black and white panels show correlations above the threshold of 0.65 MI.

**Figure 6:**
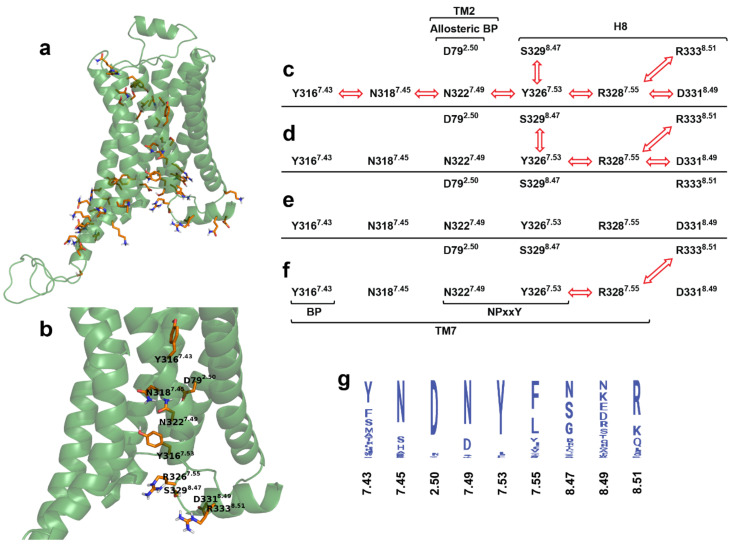
The polar signaling channel of the β_2_AR identified by dynamic cross-correlation analysis. (**a**) Polar amino acids of which motions are correlated in the G_s_ protein-bound active state. (**b**) Polar amino acids of which motions are correlated and connecting the orthosteric binding pocket to the G protein-binding interface. Non-polar hydrogens are omitted for clarity. (**c**) Active β_2_AR–G_s_ protein–epinephrine complex, 1st replica; (**d**) inactive β_2_AR–G_s_ protein–epinephrine complex; (**e**) active β_2_AR–β-arrestin-2–epinephrine complex; (**f**) inactive β_2_AR–β-arrestin-2–epinephrine complex. Red arrows indicate correlated motions of the respective amino acids. (**g**) Degree of conservation of polar signaling channel residues of human class A GPCRs.

Dynamic cross-correlation matrix (DCCM) analysis (Figure 5, Figure S14) of the transmembrane domain and the extra- and intracellular loops indicated, that similar to that observed for the MOP receptor previously [27], the orthosteric binding pocket and the G protein-binding interface of the β_2_AR are connected through a channel of polar amino acids which are engaged in concerted motions in the active, or constitutively active states of the receptor (Figure 6, Figure S15). However, there are several differences between the channel residues of the MOP and the β_2_AR. The first difference is that, unlike in the case of MOP, the residues of the DRY motif are not involved in correlated motions. In the active G_i_ protein-bound MOP, R165^3.50^ of the DRY and D340^8.47^ of H8 were frequently connected through a salt bridge and, consequently, their motions were in intense correlation. In β_2_AR no such salt bridge could be formed by the analogous S329^8.47^ of H8 and the occurrence of H-bonds was also very low, which may provide an explanation for the missing involvement of the DRY motif (See further discussion below). Nevertheless, S329^8.47^ was found to move in accord with the NPxxY motif in β_2_AR, but the occurrence of that connection was also not as pronounced as in the case of MOP. Instead, D331^8.49^ and R333^8.51^ residues of the H8 showed a high degree of correlation. R333^8.51^ is conserved (Figure 6) and was suggested previously to be important for the G protein-coupling of the adenosine A_2B_ receptor [38] While such occasional similarities with other class A GPCRs may support the importance of H8 residues, their increased variability compared to the other residues of the channel could also be associated with G protein specificity. A further difference observed between the MOP and β_2_AR is that the allosteric Na^+^ binding site (D79^2.50^) is less intensely involved in the correlated motions of channel residues of β_2_AR. However, this correlation was present during the reference simulation when epinephrine was mildly restrained to the orthosteric binding pocket, suggesting that the presence of a strongly bound, correctly oriented ligand initiates coupling of D79^2.50^ to the signaling cascade (Figure S15). An interesting, specific feature of the polar signaling channel of β_2_AR is that it could be subdivided into two segments, suggested by the results of NPxxY disposition and DCCM analysis. The first segment spans the orthosteric (Y316^7.43^) and allosteric (D79^2.50^) binding pockets, N318^7.45^ and the NPxxY motif (N322^7.49^ and Y326^7.53^), while the second segment shares the last residue of the NPxxY motif (Y326^7.53^) and includes the tip of TM7 (R328^7.55^) and the three H8 residues (S329^8.47^, D331^8.49^, and R333^8.51^) (Figure 6, Figure S15). The rationale behind this subdivision is provided by NPxxY disposition data as was already mentioned earlier in this report. The relatively large, approximately 0.4 nm (RMSD) disposition of the NPxxY motif resulted in intense concerted motions in the above-mentioned second segment of the polar signaling channel. Such movements were observed in inactive states and in the absence of ligand, whereas the full sequence of correlated motions was incomplete in those systems. This suggests that the elevated concerted dynamics of the second segment could also be associated with the constitutive activity of β_2_AR. This presumption was supported by that correlated motions of this second segment were decoupled upon β-arrestin-2 binding and/or if epinephrine was bound in the wrong relative orientation, stabilizing a conformational state which is inappropriate for signaling. Further support is provided by results of a previous extensive study, where an intermediate structure was identified in the absence of ligands, which may represent a receptor conformation that facilitates G_s_ protein insertion and suggests that the activation process, in terms of structural changes, starts at the intracellular side of the receptor. The role of the NPxxY motif in the formation of such intermediate was also emphasized [17].

### 2.5. Intramolecular Interactions

The frequencies of intramolecular salt bridges and H-bonds, previously proposed to be relevant for class A GPCR activation, are summarized in Table 2. As opposed to observations taken previously for the MOP [27], intramolecular interactions between the DRY motif and H8 were missing from all β_2_AR systems examined here. Salt bridge formation is not facilitated between R131^3.50^ and S329^8.47^ and no other potential, proximal partners were found in H8 that could participate in the formation of a salt bridge analogous to that between R165^3.50^ of the DRY and D340^8.47^ of H8 in the active, G_i_ protein-bound MOP. No stable H-bond formation was indicated between the DRY motif and H8 either. This specific interaction was first described in our previous report [27] as it was not present in the high-resolution experimental structures of the MOP. Since this interaction is not facilitated in β_2_AR, it is most likely a specific property of the MOP. Considerable frequencies of salt bridges and H-bonds were observed between the neighboring D130^3.49^ and R131^3.50^ of the DRY motif in the active states, although high-resolution experimental structures indicated coincidentally that this interaction is only present in inactive states and absent in active receptors [5,7,8,11,12,13]. Formation of the “ionic lock” between the DRY motif (R131^3.50^) and TM6 (E268^6.30^) was also not observed, neither in the active nor in the inactive states. According to earlier proposals, this interaction acts as a constraint in the inactive state and gets disrupted upon receptor activation, followed by the release and disposition of TM6 [21]. Mutations affecting the participants of this ionic lock resulted in the elevated constitutional activity of the β_2_AR [39], however, the presence of this ionic lock was not corroborated by the crystallographic structures of this receptor [5,12]. The absence of ionic lock interactions in these structures was attributed to residual basal activity present in the crystalline state [12]. Formation of this ionic lock was observed previously in MD simulations of receptor deactivation, but in the absence of intracellular signaling proteins and at significantly longer timescales [17].

In agreement with the experimental structure [7], the systematic presence of H-bonds between D130^3.49^ of the DRY motif and Y141 of ICL2 was indicated in simulations of the active state β_2_AR. In inactive states, D130^3.49^ of the DRY motif was found to interact with S143 and L144. This is in agreement with the results of the secondary structure analysis of ICL2 and supports the discussion above, regarding the role of this loop in the activation mechanism. No considerable trends were observed between the different receptor states and the frequencies of DRY-TM5 [40], CWxP-TM7 [41], and D113^3.32^-Y316^7.43^ interactions [40] within the time frame of simulations.

### 2.6. Intermolecular Interactions

The results of analyses of intermolecular interactions are summarized in Table 3. Similar to that observed previously for the MOP receptor [27], the interaction between the ligand and the anchor residue of the binding pocket (D113^3.32^) was strongest in the active, G_s_ protein-bound β_2_AR. The difference between active, inactive, G_s_ protein, and β-arrestin-2 bound states was not as outstanding as in the case of MOP. This observed trend is also in slight contradiction with the fact that ligand disposition also occurred in G_s_ protein-bound active states. Analysis of β_2_AR–G_s_ protein interactions demonstrated that contacts between ICL1 and helix 5 of the α subunit of the G_s_ protein (H5Gα) are negligible in ligand-bound receptors, regardless of the activation state. Medium frequency was, however, observed in ligand-free states. The ICL2-H5Gα contact was expected to be the most specific among the contacts between the G_s_ protein and the β_2_AR [7], based on previous results [27] and the above presented secondary structure and intramolecular H-bond analysis. Even so, this contact was found to be very weak during simulations of the active state, epinephrine and G_s_ protein-bound β_2_AR. Higher frequencies of H-bonds between ICL2 and H5Gα were observed in inactive states and the absence of epinephrine. This may suggest that the loss of interaction between ICL2 and H5Gα in agonist-bound active states indicates the initiation of G_s_ protein dissociation, although longer simulations would be needed to confirm this assumption. The frequency of ICL3-H5Gα H-bonds was found to be high in all simulation setups, therefore, a specific role of this contact cannot be deduced from the results presented here. Differences were found in the interactions between β-arrestin-2 and β_2_AR, depending on the presence of epinephrine. In the active ligand-bound state the finger loop (FL) of β-arrestin-2 was found to be in frequent contact with H8, ICL1, and ICL3, whereas in the active ligand-free state FL was in stronger contact with ICL2 at the expense of contacts with ICL3. In the inactive states, a higher preference of FL towards ICL1 was observed, regardless of the presence of epinephrine. No contacts were found between ICL3 of the agonist-bound β_1_ adrenergic receptor (β_1_AR) and the finger loop of β-arrestin-1 in a recent cryo-electronmicroscopic (cryo-EM) structure of this molecular complex [42]. However, a parallel study of the neurotensin receptor 1 (NTSR1)–β-arrestin-1 complex revealed, that the interface between β-arrestin-1 and NTSR1, including the finger loop, is highly dynamic and the relative orientations captured by the cryo-EM structure are likely to represent one of many conformational states [43]. A specific contact between the C-loop of β-arrestin-2 (CL) and the unfolded ICL2 was indicated in the inactive ligand-bound receptor. In the inactive ligand-free receptor ICL2 was rather in contact with the middle loop of β-arrestin-2 (ML), but with significantly lower frequency. The overall frequency of interactions was highest for inactive, epinephrine-bound β_2_AR suggesting that it is the most preferred for β-arrestin-2 binding. However, taking into account that the ligand dissociated during the corresponding simulation, such an assumption cannot be taken. The second highest H-bond frequency between β_2_AR and β-arrestin-2 was observed for the active, epinephrine-bound state. This latter apparent preference is corroborated by experimental data reporting the visual arrestin-bound [44] and active, G_T_ protein-bound structures of rhoposhin [45], which were almost identical. On the other hand, the cryo-EM structure of β_1_AR and β-arrestin-1 demonstrated that this receptor adopts an intermediate state with regard to the disposition of TM6 when bound by β-arrestin-1 [42].

## 3. Methods

### 3.1. System Building

The sequence of the human β_2_AR (UniProtKB-P07550-ADRB2) was obtained from the UniProt database (http://www.uniprot.org, accessed on 23 August 2021). All X-ray crystallographic structures used in this study were downloaded from the Brookhaven Protein Data Bank (http://www.rcsb.org, accessed on 23 August 2021). The active and inactive state β_2_AR (pdb codes: 3SN6 and 2RH1, respectively) [5,7], β-arrestin-2 (pdb code: 3P2D) [44], the G_s_α protein (pdb code: 1AZT) [46] and the β_2_AR-bound epinephrine (pdb code: 4LDO) [8] were used as starting structures for MD simulations in this study. All crystallographic chaperones and fusion proteins were removed from the corresponding structures. The α subunit of the G_s_ protein complex was missing from the crystallographic structure of the active β_2_AR (pdb code: 3SN6) [7], therefore, it was supplemented from an independent crystallographic structure of that subunit (pdb code: 1AZT) [46], together with the bound GDP. Epinephrine was inserted in the binding pocket of the receptors in a protonated form. The third intracellular loop (ICL3, E237-K267) of β_2_AR, missing from the crystallographic structures was modeled using the Modeller ver. 9.20 software [47] and the missing residues in the second extracellular loop (ECL2, A176-H178) were retrieved using the Swiss-PdbViewer ver. 4.10 program [48]. The missing *N*- and *C*-terminal domains (M1-D29 and C341-L413, respectively) of β_2_AR were modeled by performing 10 ns folding simulations using the GROMACS ver. 2018.3 program package [49], following a previously described protocol [27] and attached to the TM domain of the receptor manually.

The CHARMM-GUI [50] web-based platform was used to include post-translational modifications of β_2_AR and to insert the receptor in a solvated membrane bilayer. Residues N6, N15, and N187 were glycosylated [51] and residue C341 was palmitoylated in both the active and inactive state receptors [52], whereas phosphorylations at the *C*-terminal domain (S355, S356, and S364) [53,54] were included only for β-arrestin-2 bound systems. Complex type glycans were used for glycosylation of the *N*-terminal domain, consisting of a common core (Manα1–3 (Manα1–6) Manβ1–4GlcNAcβ1–4GlcNAcβ1–*N*) and sialic acid (*N*-acetylneuraminic acid).

The receptor complexes were inserted in a previously introduced and examined explicit caveolar membrane bilayer [27] using the membrane builder tool of CHARMM-GUI [50]. The bilayer consisted of the following components: cholesterol (CHL-32.8%), 1-palmitoyl-2-oleoyl-glycero-3-phosphocholine (POPC-14.9%), 1-palmitoyl-2-oleoyl-sn-glycero-3-phosphoethanolamine (POPE-27.8%), 1-palmitoyl-2-oleoyl-sn-glycero-3-phospho-L-serine (POPS-3.6%), 1-palmitoyl-2-oleoyl-sn-glycero-3-phosphoinositol (POPI2–6.0%), palmitoyl-sphingomyelin (PSM-9.9%), and monosialodihexosylganglioside (GM3–5.0%) [55]. The asymmetric lower and upper leaflet compositions of the membrane were set according to the recent literature data [56]. Membrane-inserted β_2_AR complexes were then solvated with explicit TIP3P water molecules in a hexagonal-shaped periodic box, and Na^+^ and Cl^−^ ions (0.15 M) were added to neutralize the system and to attain physiological ionic strength. System coordinates and topologies were generated in GROMACS format and CHARMM36 all-atom force field parameters were assigned to all system components [57].

### 3.2. MD Simulations

All energy minimizations and MD simulations were performed using the GROMACS 2018.3 program package [49]. Initially, all complex systems were subjected to 5000 steps steepest descent, and then 5000 steps conjugate gradient energy minimization. The convergence criteria were set to 1000 kJ mol^−1^ nm^−1^ for both minimization steps. Minimized systems were then thoroughly equilibrated following a six-step protocol, supplied by CHARMM-GUI. According to the protocol, two consecutive MD simulations were executed at 303.15 K temperature in the canonical (NVT) ensemble, then four further simulations in the isobaric (NPT) ensemble at 303.15 K temperature and 1 bar pressure. Positional restraints were applied on the heavy atoms of the proteins and membrane constituents which were decreased gradually throughout the steps of the equilibration protocol. The first three equilibration MD runs were 25 ps long and were performed in 1 fs time steps. The following two were continued for 100 ps in 2 fs time steps. The last equilibration step was extended to 50 ns and was executed in 2 fs time steps. The chemical bonds were constrained to their correct lengths using the LINCS algorithm. The v-rescale algorithm [58] with a coupling constant of 1 ps was applied for temperature control. The pressure was regulated using Berendsen (semi-isotropic) pressure coupling [59] with a 5 ps coupling constant and 4.5 × 10^−5^ bar^−1^ isothermal compressibility. The Particle Mesh Ewald (PME) method was used to calculate energy contributions from electrostatic interactions. Van der Waals interactions were calculated using a twin-range cutoff. All cut-off values were set to 1.2 nm.

Eleven independent production simulations were performed at 310 K in the NPT ensemble, with other parameters similar as above. Each simulation was 1 μs long and included the active and inactive state β_2_AR, complexed either with the heterotrimeric G_s_ protein or β-arrestin-2, in the presence or the absence of orthosterically-bound epinephrine. The simulation of the active β_2_AR bound to the G_s_ protein and epinephrine was performed in three replicates. An additional reference simulation was performed for this system in which a mild positional restraint (200 kJ mol^−1^ on heavy atoms) was applied for epinephrine to prevent spontaneous ejection from the binding pocket. None of the other production simulations have included any restraints. System coordinates were saved after every 5000 steps providing trajectories with 100,000 snapshots.

### 3.3. MD Trajectory Analysis

MD trajectories were examined using the analysis suite of the GROMACS 2018.3 package [49] to check the integrity of simulation systems and to observe protein conformational changes and minute details of different activation states of the β_2_AR. Specific analyses were performed to compare results to those obtained for the MOP in a recent study [27].

Root mean square deviation (RMSD) of protein backbone atoms were calculated and compared between different systems to assess the structural stability of the macromolecular complexes and to identify significant displacements of key structural components as a function of time. The gmx rms program was used for RMSD calculation. The gmx helix utility was used to calculate properties of TM helices and to measure their deviation from the ideal α-helical structure. Secondary structures of intracellular loops (ICL1: F61-T66, ICL2: S137-T146, ICL3: E237-K267), and the cytosolic helix (H8: S329-L340) was assigned using the DSSP method [60]. The frequency of intra- and intermolecular H-bonds was calculated using the gmx hbond program. H-bonds were assigned within 0.35 nm donor-acceptor distance and below 30.0 degrees of donor-hydrogen-acceptor angle. The presence of salt bridges between acidic and basic functional groups was assigned with 0.40 nm distance and 90.0 degrees angle cutoff values. Distances and angles between these groups were calculated with the gmx distance and gmx gangle programs, respectively. The gmx mindist program was used to observe Na^+^ penetration into the TM domain and to calculate the minimum distance between periodic replicas of *N*- and *C*-terminal domains. Dynamic cross-correlation matrix (DCCM) analysis, available in an earlier version of the GROMACS suite (g_correlation, ver. 3.3) [61], was performed to examine the dynamic motions of amino acid side chains in the TM domain and connecting loops. DCCM matrices were converted to heat map images using the gmx xpm2ps utility and analyzed using the Gimp ver. 2.8 software. Color intensities corresponding to 0.65 MI (mutual information) and the participation of at least four atoms from each amino acid side chain were set as the threshold of correlation assignment. The Pymol ver. 2.1.0 and VMD 1.9.4a12 software were used for molecular visualization. The Xmgrace ver. 5.1.25. program was used to prepare graphs.

### 3.4. Sequence Alignment and Conservation Analysis

Downloaded from the UniProt database in FASTA format were 267 sequences of class A human GPCRs (without orphan and olfactory receptors). Multiple-sequence alignment was carried out using the Clustal Omega program [62] and analyzed with the Jalview 2.10.5 software [63]. The ADRB2_HUMAN (P07550) was set as the reference sequence for conservation analysis.

## 4. Conclusions

Results presented here for the β_2_AR provide support for our previously proposed hypothesis [27] and justify its extension to other class A GPCRs. The above results also suggest that the previously proposed potential contribution of the electrostatic balance in the TM domain is warranted for detailed, quantitative examination. While several previous assumptions drawn from results gathered for the MOP receptor were reinforced, some had to be adjusted to account for the differences observed in the case of β_2_AR. The general features of GPCR activation proposed here and previously by others [16,20,25,26] and the receptor-specific, characteristic details may provide alternative opportunities for the discovery of a new class of GPCR drugs. The extended perspective of the activation mechanism, if further pursued, may provide a more in-depth explanation for ligand-induced effects in multiple functional states and could help to identify and quantitatively assess specific physico-chemical properties of GPCR ligands that furnish different functional properties.

## Figures and Tables

**Table 1:** The frequency of contact (d ≤ 0.4 nm) between Na^+^ ions and polar amino acid side chains of the allosteric and orthosteric binding pockets and nearby conserved motifs.

Residue	Frequency of Contact/%							
	Epinephrine-Bound β_2_AR				Ligand-Free β_2_AR			
	G_s_ Protein Complex		β-Arrestin-2 Complex		G_s_ Protein Complex		β-Arrestin-2 Complex	
	Active	Inactive	Active	Inactive	Active	Inactive	Active	Inactive
D_79_^2.50^	0.0	0.0	0.0	0.0	52.15	93.41	25.91	26.04
D_113_^3.32^	<0.1	32.33	0.0	0.0	9.22	45.06	5.02	72.03
S_120_^3.39^	0.0	0.0	0.0	0.0	44.95	96.36	5.55	25.86
W_286_^6.48^	0.0	6.88	0.0	0.0	3.36	19.51	29.16	10.92
N_318_^7.45^	0.0	0.0	0.0	0.0	2.78	0.46	46.14	0.22
S_319_^7.46^	0.0	0.0	0.0	0.0	30.52	14.39	79.75	24.69
N_322_^7.49^	0.0	0.0	0.0	0.0	45.87	51.12	8.17	20.06
Y_326_^7.53^	0.0	0.0	0.0	0.0	0.12	0.60	0.0	0.0

**Table 2:** Frequency of intramolecular salt bridges and H-bonds expressed as percentages of the total conformational ensemble, generated by MD simulations.

Interactions	Residues Involved	Epinephrine-Bound							Ligand-Free			
		Active State					Inactive State		Active State		Inactive State	
		G_s_ Protein Complex				β-Arrestin-2	G_s_ Protein Complex	β-Arrestin-2	G_s_ Protein Complex	β-Arrestin-2	G_s_ Protein Complex	β-Arrestin-2
		1	2	3	Restrained							
Salt Bridge												
Intra-DRY	D130^3.49^; R131^3.50^	26.5	5.7	6.0	0	65.1	40.4	35.5	40.5	10.7	51.2	41.3
H-bonds												
DRY-H8	R131^3.50^; S329^8.47^	0.1	0	0.3	0	0.1	0	0	0.1	0.1	0.0	0.0
BP	D113^3.32^; Y316^7.43^	14.0	80.6	82.1	95.3	28.3	65.9	69.2	96.1	9.9	0.1	14.6
intra-DRY	D130^3.49^; R131^3.50^	65.9	64.5	17.9	16.8	35.7	80.0	72.9	82.3	30.5	99.8	83.5
DRY-ICL2	D130^3.49^; Y141^ICL2^	99.3	97.5	92.4	98.0	99.8	0	0	98.1	86.2	0	0
	D130^3.49^; S143^ICL2^; L144^ICL2^	0	0	0	0	0	99.5	90.8	0	0	0	37.3
DRY-TM5	R131^3.50^; Y219^5.58^	3.9	10.5	4.6	45.2	30.9	0	0	5.7	3.3	0	0
DRY-TM6	R131^3.50^; E268^6.30^	0	0	0	0	0	0	0	0	0	0	0
CWxP-TM7	C285^6.47^; W286^6.48^; N318^7.45^	1.8	3.4	45.1	4.1	5.3	11.7	4.3	17.6	0.5	53.7	3.8

BP = orthosteric binding pocket of the β_2_AR; Ballesteros–Weinstein numbering of residues is indicated in superscript.

**Table 3:** Frequency of intermolecular salt bridges and H-bonds expressed as percentages of the total conformational ensemble, generated by MD simulations.

Interactions	Residues Involved, Respectively	Epinephrine-Bound							Ligand-Free			
		Active State					Inactive State		Active State		Inactive State	
		G_s_ Protein Complex				β-Arrestin-2	G_s_ Protein Complex	β-Arrestin-2	G_s_ Protein Complex	β-Arrestin-2	G_s_ Protein Complex	β-Arrestin-2
		1	2	3	Restrained							
Salt Bridge												
BP-epi	D113^3.32^; epi	67.5	51.0	4.8	52.7	36.0	29.3	60.1	-	-	-	-
H-bonds												
BP-epi	D113^3.32^; epi	96.7	96.0	8.3	99.3	55.5	53.8	93.3	-	-	-	-
H8-H5Gα	S329-L340; T369-L394	0.0	7.3	4.0	0.0	-	0.0	-	0.0	-	0.0	-
ICl1-H5Gα	F61-T66; T369-L394	0.2	0.1	0.0	0.2	-	0.6	-	30.3	-	40.0	-
ICl2-H5Gα	S137-T146; T369-L394	0.1	0.1	17.9	0.5	-	9.9	-	5.1	-	31.5	-
ICL3-H5Gα	E237-K267; T369-L394	90.5	92.5	97.5	96.3	-	77.1	-	76.2	-	76.7	-
H8-FL	S329-L340; G65-K78	-	-	-	-	33.4	-	0.0	-	0.0	-	0.0
ICl1-FL	F61-T66; G65-K78	-	-	-	-	25.6	-	58.8	-	22.9	-	49.3
ICL2-FL	S137-T146; G65-K78	-	-	-	-	8.7	-	30.4	-	77.8	-	35.6
ICL3-FL	E237-K267; G65-K78	-	-	-	-	85.5	-	71.7	-	14.8	-	14.0
H8-ML	S329-L340; P132-A140	-	-	-	-	0.0	-	0.0	-	0.0	-	0.0
ICL1-ML	F61-T66; P132-A140	-	-	-	-	0.9	-	0.1	-	0.0	-	0.0
ICL2-ML	S137-T146; P132-A140	-	-	-	-	7.1	-	4.2	-	9.3	-	30.3
ICL3-ML	E237-K267; P132-A140	-	-	-	-	0.0	-	0.0	-	0	-	0
H8-CL	S329-L340; V307-G317	-	-	-	-	0.0	-	0.0	-	0.0	-	0.0
ICl1-CL	F61-T66; V307-G317	-	-	-	-	0.0	-	0.0	-	0.0	-	0.0
ICL2-CL	S137-T146; V307-G317	-	-	-	-	4.6	-	58.4	-	0.2	-	8.9
ICL3-CL	E237-K267; V307-G317	-	-	-	-	0.0	-	0.0	-	4.0	-	0

BP = orthosteric binding pocket of the β_2_AR; epi = epinenphrine; H5Gα = helix 5 of the G_s_ protein α subunit; FL/ML/CL = finger loop/middle loop/C loop of β-arrestin-2. Ballesteros–Weinstein numbering of residues is indicated in superscript.

## Data Availability

All data contained within the article or Supplementary Materials are available upon request.

## Supplementary Materials

The Supplementary Materials are available online at https://www.mdpi.com/article/10.3390/ijms221910423/s1.
