# Supplementary material for: Universal Properties and Specificities of the β2-Adrenergic Receptor-Gs Protein Complex Activation Mechanism Revealed by All-Atom Molecular Dynamics Simulations
Source: Int J Mol Sci. 2021 Sep 27;22(19):10423. doi: 10.3390/ijms221910423 (PMC8508748; doi:10.3390/ijms221910423)
Supplement: Supplementary file 1 [file ijms-22-10423-s001.zip › ijms-1380727-supplementary.pdf]

International Journal of
Molecular Sciences

MDPI

Article

Universal Properties and Specificities of the β_2-Adrenergic Receptor-G_s Protein Complex Activation Mechanism Revealed by All-Atom Molecular Dynamics Simulations.

Argha Mitra [1,2], Arijit Sarkar [1,2], Attila Borics [1,*]

[1] Laboratory of Chemical Biology, Institute of Biochemistry, Biological Research Centre, Szeged, 62. Temesvári krt., H-6726 Szeged, Hungary; argha.mitra@brc.hu (A.M.); sarkar.arajit@brc.hu (A.S.)
[2] Theoretical Medicine Doctoral School, Faculty of Medicine, University of Szeged, 97. Tisza L. krt., H-6722 Szeged, Hungary
* Correspondence: borics.attila@brc.hu, Phone: +36 62 599 600 ext. 430

Supplementary materials

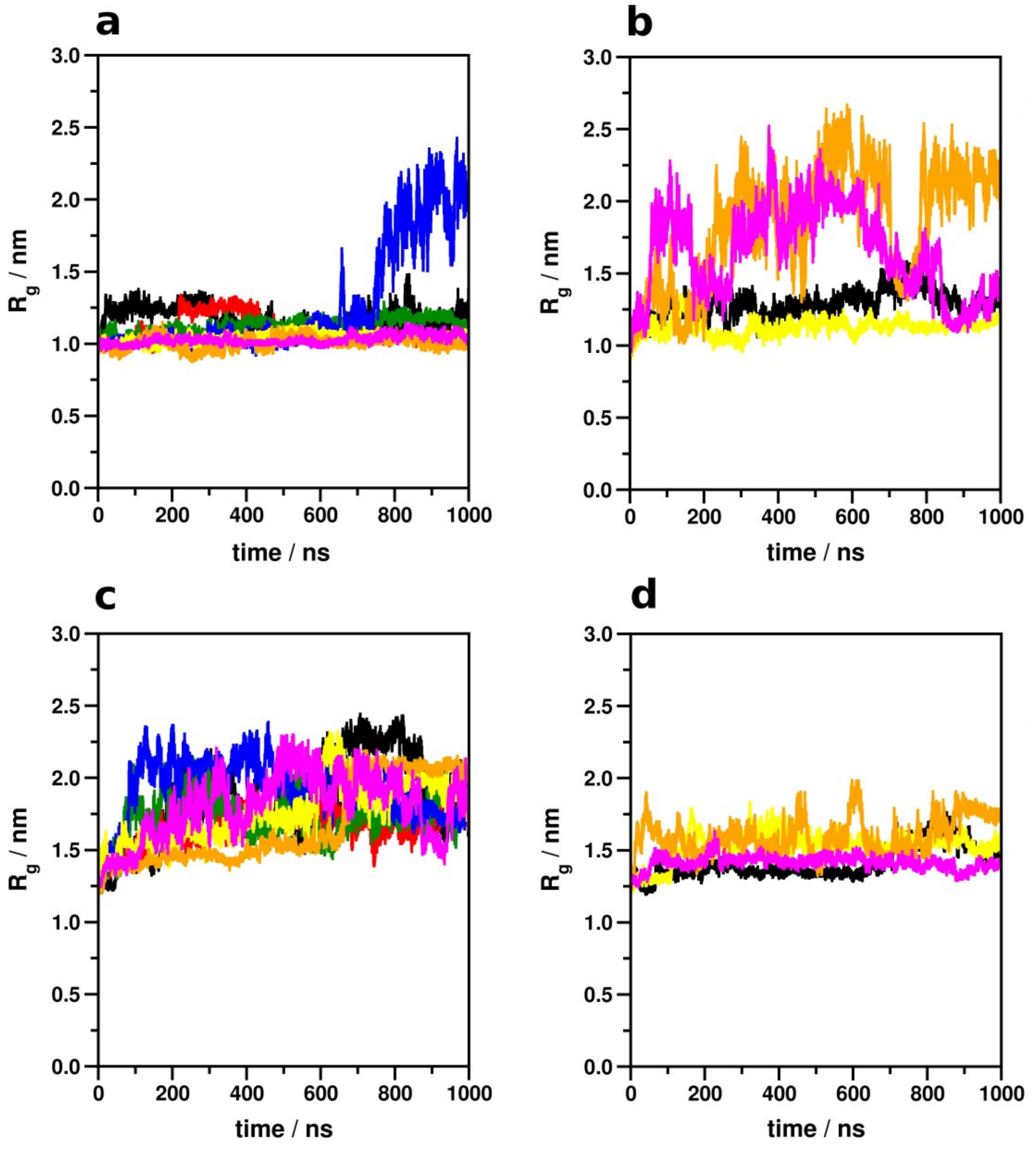

Figure S1. The evolution of radii of gyration of the N- (**a**,**b**) and C-terminal domains (**c**,**d**) of the active (**a**,**c**) and inactive state (**b**,**d**) β2AR during production simulations. Black: β2AR – Gs protein – epinephrinecomplex, 1st replica; Red: β2AR – Gs protein – epinephrine complex, 2nd replica; Green: β2AR – Gs protein – epinephrine complex, 3rd replica; Blue: β2AR – Gs protein – epinephrine complex, with restrained epinephrine and GDP; Yellow: β2AR – β-arrestin-2 – epinephrine complex; Orange: ligand-free β2AR – Gs protein complex; Magenta: ligand-free β2AR – β-arrestin-2 complex.

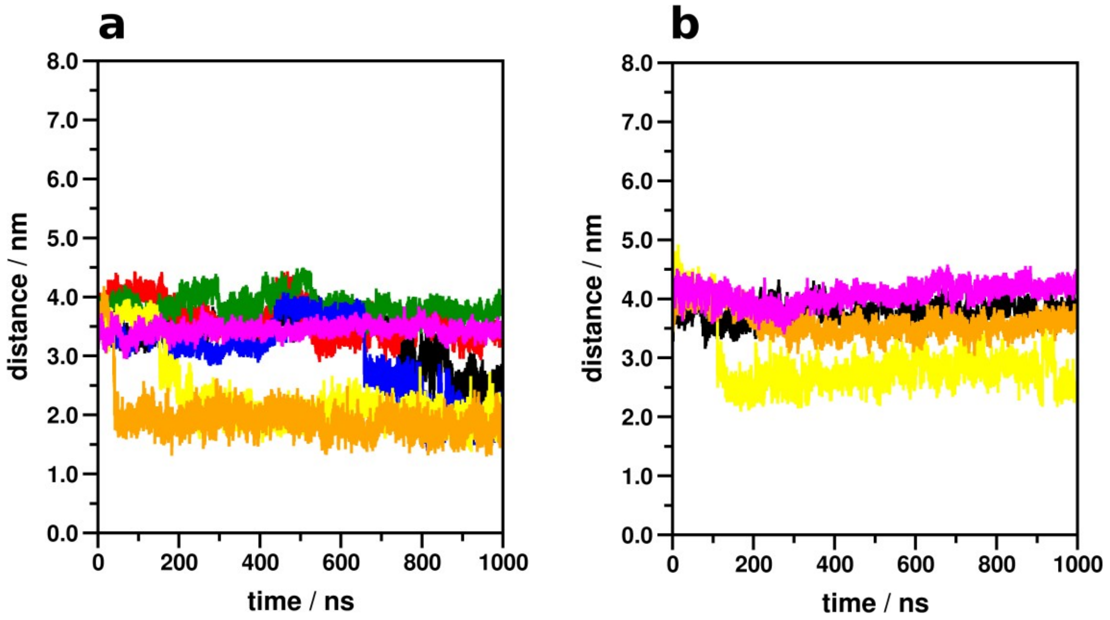

Figure S2. Minimum distance between the *N*- and *C*-terminal domains (and their periodic replicas) of the active (**a**) and inactive state (**b**) β2AR during production simulations. Black: β2AR – Gs protein – epinephrine complex, 1st replica; Red: β2AR – Gs protein – epinephrine complex, 2nd replica; Green: β2AR – Gs protein – epinephrine complex, 3rd replica; Blue: β2AR – Gs protein – epinephrine complex, with restrained epinephrine and GDP; Yellow: β2AR – β-arrestin-2 – epinephrine complex; Orange: ligand-free β2AR – Gs protein complex; Magenta: ligand-free β2AR – β-arrestin-2 complex.

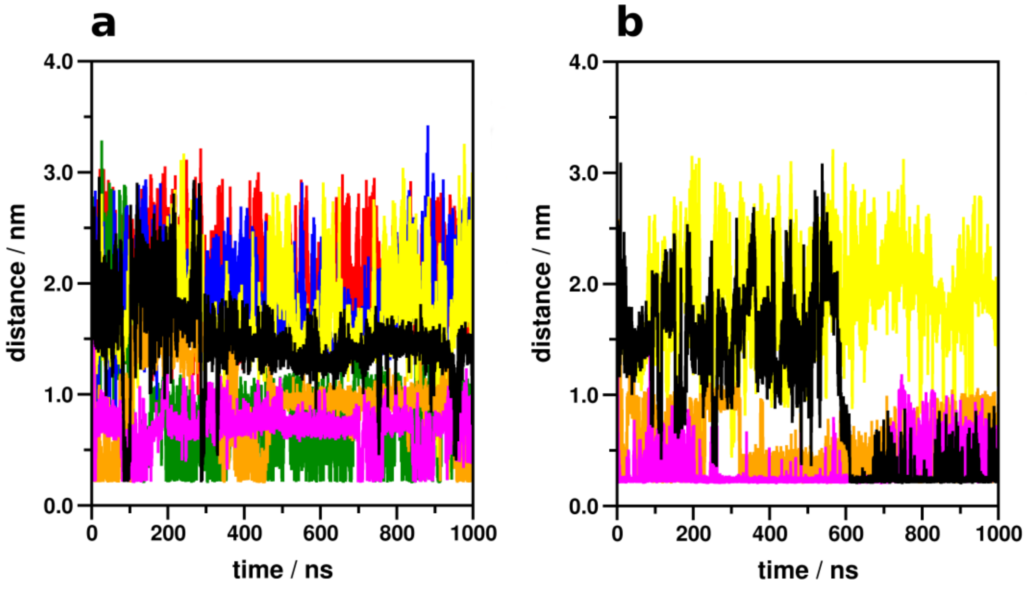

Figure S3. Minimum distance between Na^+ ions and D113$^{3.32}$ of the orthosteric binding pocket of the active (**a**)and inactive state (**b**) β2AR during simulations. Black: β2AR – Gs protein – epinephrine complex, 1st replica; Red: β2AR – Gs protein – epinephrine complex, 2nd replica; Green: β2AR – Gs protein – epinephrine complex, 3rd replica; Blue: β2AR – Gs protein – epinephrine complex, with restrained epinephrine and GDP; Yellow: β2AR – β-arrestin-2 – epinephrine complex; Orange: ligand-free β2AR – Gs protein complex; Magenta: ligand-free β2AR – β-arrestin-2 complex.

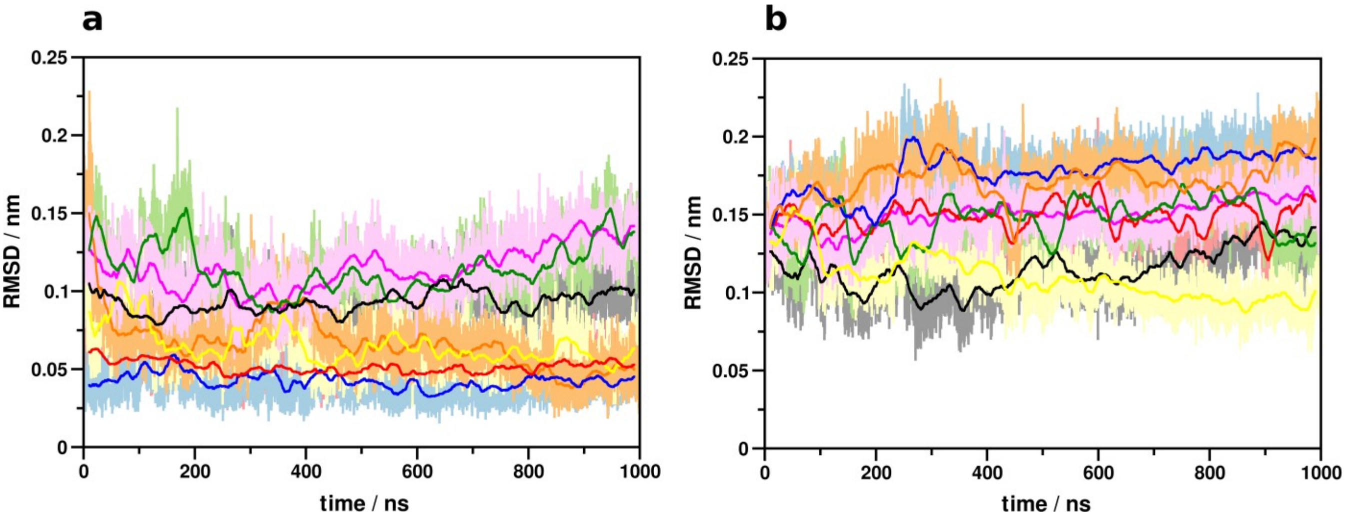

Figure S4. Properties of transmembrane helices. (**a**) Deviation from ideal α-helical geometry in the Gs protein and epinephrine-bound active state. Black: TM1, red: TM2, green: TM3, blue: TM4, yellow: TM5, orange: TM6, magenta: TM7. (**b**) Deviation of TM7 from ideal ⊚-helical geometry in the active, Gs protein and epinephrine-bound (black: 1st replica; red: 2nd replica; green: 3rd replica; blue: restrained epinephrine and GDP), active, β-arrestin-2 and epinephrine-bound (yellow), active, Gs protein-bound, ligand free (orange) andactive, β-arrestin-2-bound, ligand-free states (magenta).

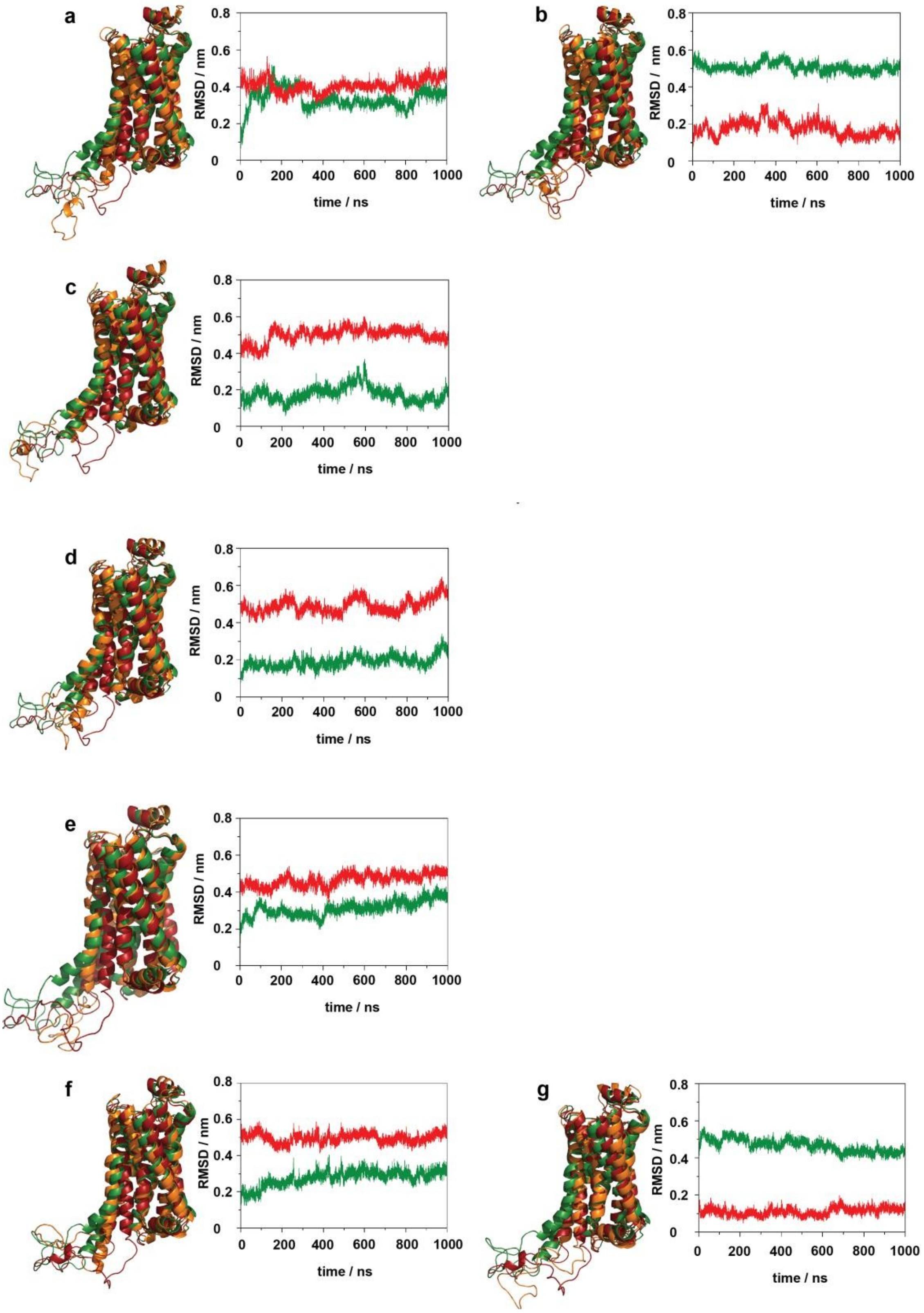

Figure S5. Disposition of TM6 during simulations (orange) with respect to the active (green) and inactive (red) crystallographic structures of the β_2AR. (**a**) active β_2AR – G_s protein – epinephrine complex, 1st replica; (**a**)inactive β_2AR – G_s protein – epinephrine complex; (**c**) active β_2AR – G_s protein – epinephrine complex, 2nd replica; (**d**) active β_2AR – G_s protein – epinephrine complex, 3rd replica; (**e**) active β_2AR – G_s protein – epinephrine complex, with restrained epinephrine and GDP; (**f**) active β_2AR – β-arrestin-2 – epinephrine complex; (**g**) inactive β_2AR – β-arrestin-2 – epinephrine complex.

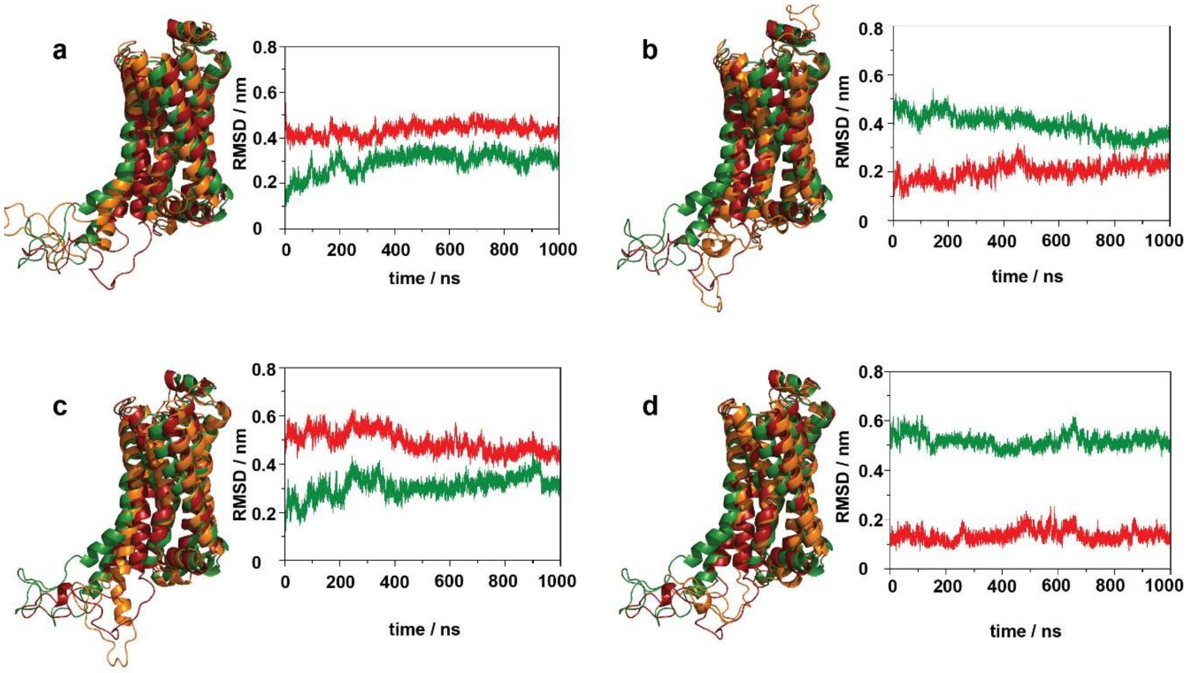

Figure S6. Disposition of TM6 during simulations (orange) with respect to the active (green) and inactive (red) crystallographic structures of the β2AR. (**a**) active, ligand-free β2AR – Gs protein complex; (**b**) inactive, ligand-free β2AR – Gs protein complex; (**c**) active, ligand-free β2AR – β-arrestin-2 complex; (**d**) inactive, ligand-free β2AR – β-arrestin-2 complex.

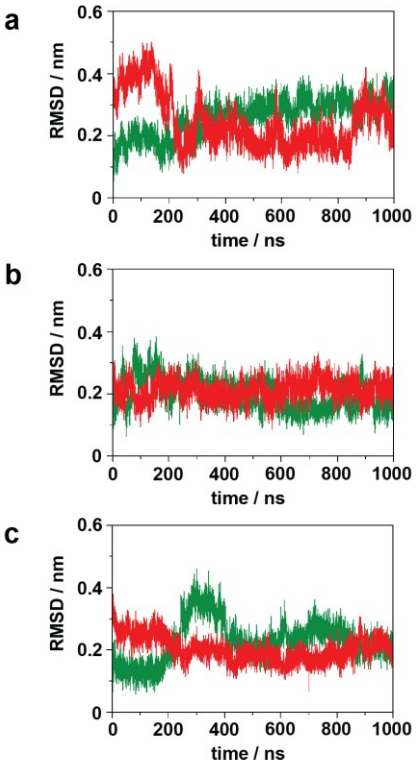

Figure S7. Disposition of the NPxxY motif during simulations with respect to the active (green) and inactive (red) crystallographic structures of the β2AR. (**a**) active β2AR – Gs protein – epinephrine complex, 2nd replica;. (**b**) active β2AR – Gs protein – epinephrine complex, 3rd replica; (**c**) active β2AR – Gs protein – epinephrinecomplex, with restrained epinephrine and GDP.

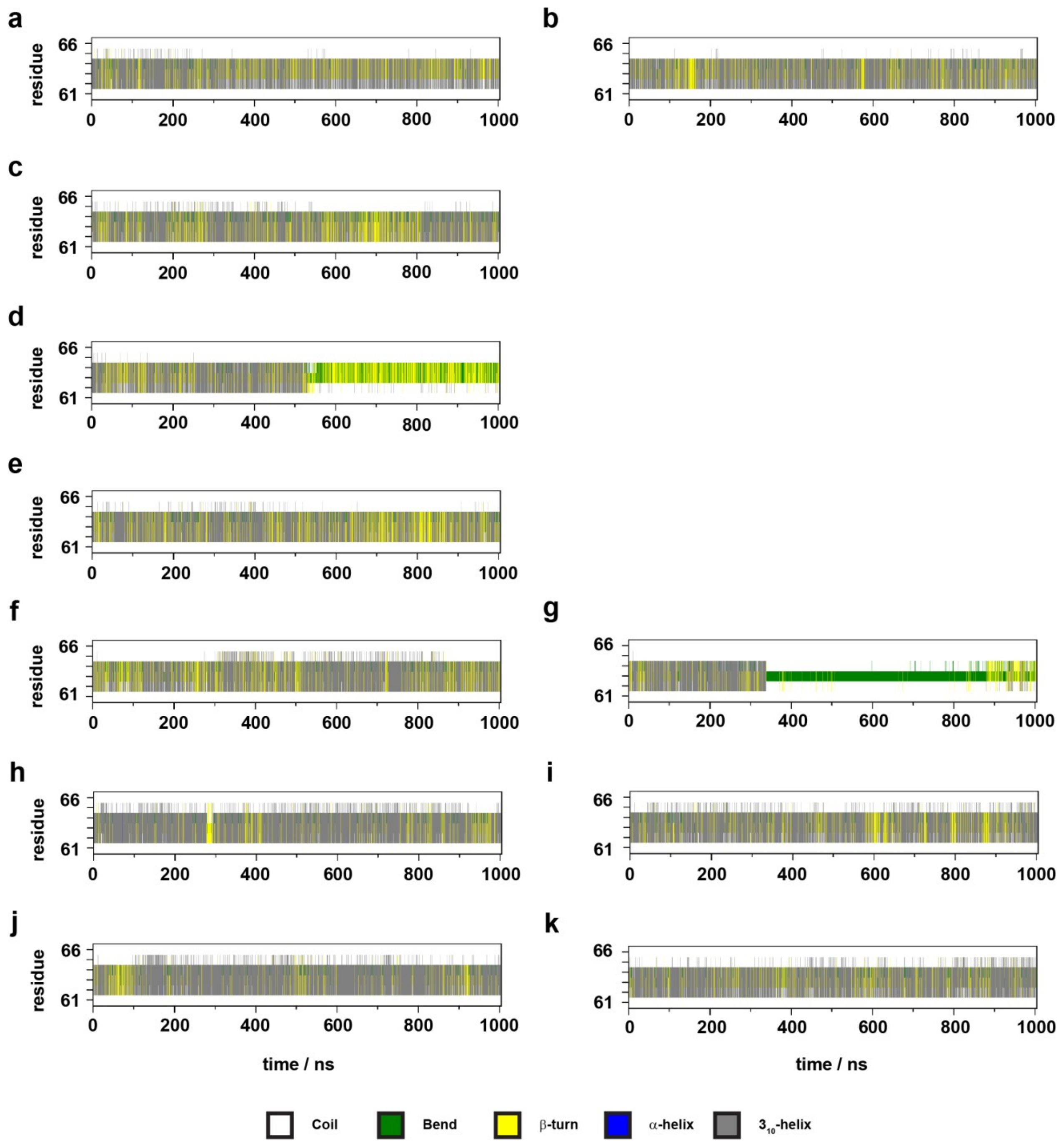

Figure S8. Evolution of the secondary structure of ICL1 during simulations. (**a**) active β2AR – Gs protein – epinephrine complex, 1st replica; (**b**) inactive β2AR – Gs protein – epinephrine complex; (**c**) active β2AR – Gs protein – epinephrine complex, 2nd replica; (**d**) active β2AR – Gs protein – epinephrine complex, 3rd replica; (**e**) active β2AR – Gs protein – epinephrine complex, with restrained epinephrine and GDP; (**f**) active β2AR – β- arrestin-2 – epinephrine complex; (**g**) inactive β2AR – β-arrestin-2 – epinephrine complex; (**h**) active, ligand- free β2AR – Gs protein complex; (**i**) inactive, ligand-free β2AR – Gs protein complex; (**j**) active, ligand-free. β2AR – β-arrestin-2 complex; (**k**) inactive, ligand-free β2AR – β-arrestin-2 complex.

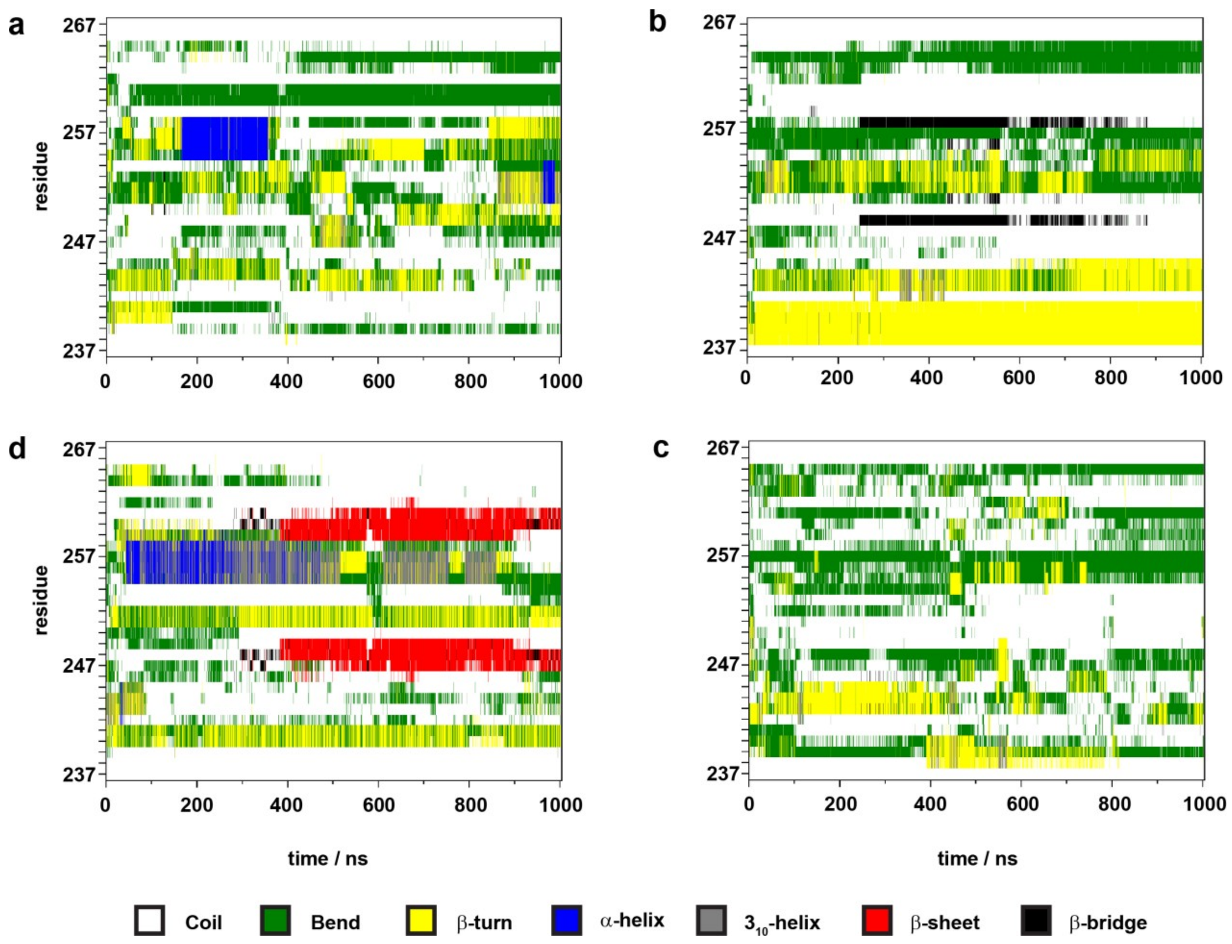

Figure S9. Evolution of the secondary structure of ICL3 during simulations. (**a**) active β2AR – Gs protein – epinephrine complex, 1st replica; (**b**) inactive β2AR – Gs protein – epinephrine complex; (**c**) active β2AR – β- arrestin-2 – epinephrine complex; (**d**) inactive β2AR – β-arrestin-2 – epinephrine complex.

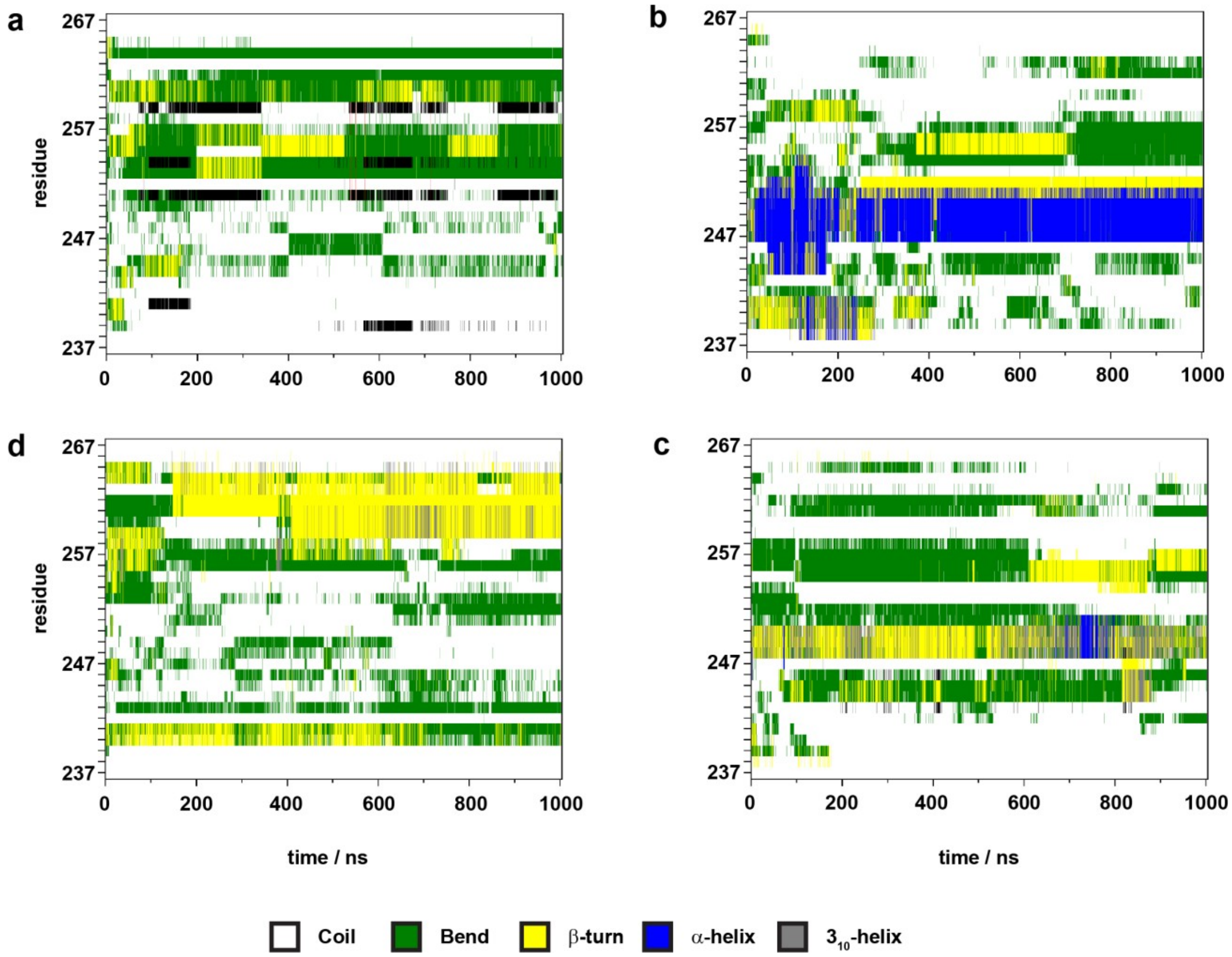

Figure S10. Evolution of the secondary structure of ICL3 during simulations. (**a**) active, ligand-free β2AR –Gs protein complex; (**b**) inactive, ligand-free β2AR – Gs protein complex; (**c**) active, ligand-free β2AR – β- arrestin-2 complex; (**d**) inactive, ligand-free β2AR – β-arrestin-2 complex.

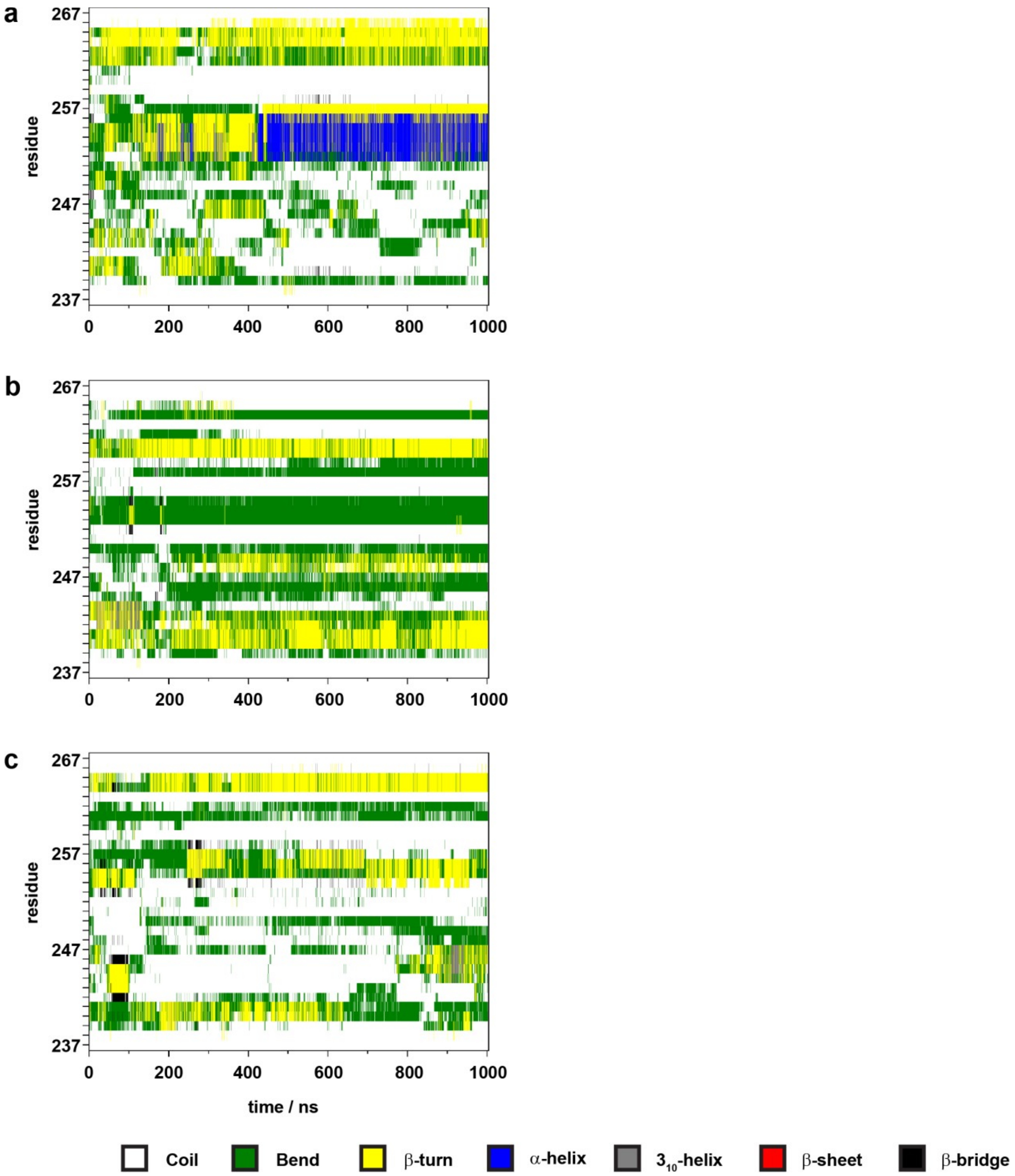

Figure S11. Evolution of the secondary structure of ICL3 during simulations. (**a**) active β2AR – Gs protein –epinephrine complex, 2nd replica; (**b**) active β2AR – Gs protein – epinephrine complex, 3rd replica; (**c**) active. β2AR – Gs protein – epinephrine complex, with restrained epinephrine and GDP.

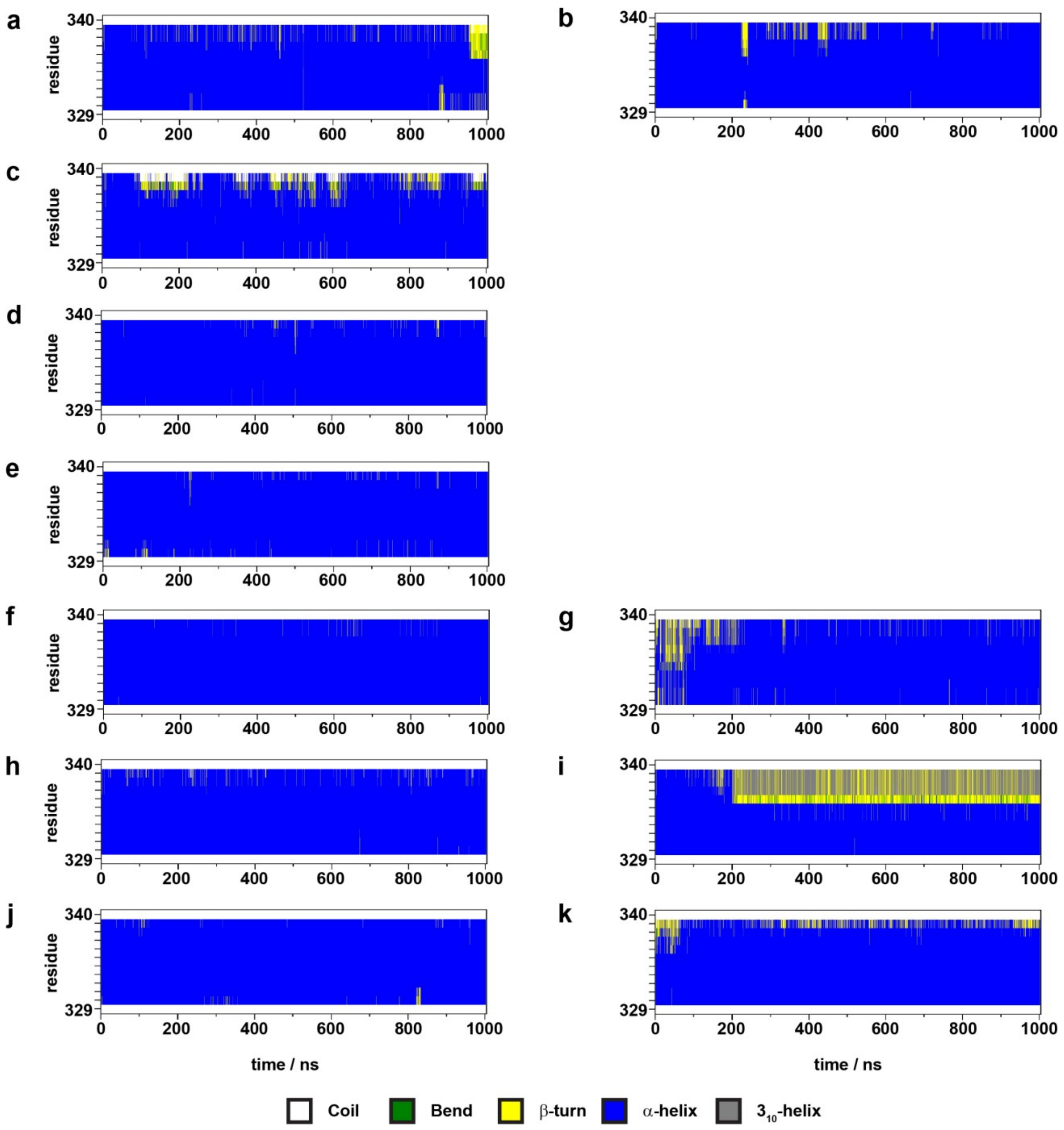

Figure S12. Evolution of the secondary structure of H8 during simulations. (**a**) active β2AR – Gs protein – epinephrine complex, 1st replica; (**b**) inactive β2AR – Gs protein – epinephrine complex; (**c**) active β2AR – Gs protein – epinephrine complex, 2nd replica; (**d**) active β2AR – Gs protein – epinephrine complex, 3rd replica; (**e**) active β2AR – Gs protein – epinephrine complex, with restrained epinephrine and GDP; (**f**) active β2AR – β- arrestin-2 – epinephrine complex; (**g**) inactive β2AR – β-arrestin-2 – epinephrine complex; (**h**) active, ligand- free β2AR – Gs protein complex; (**i**) inactive, ligand-free β2AR – Gs protein complex; (**j**) active, ligand-free. β2AR – β-arrestin-2 complex; (**k**) inactive, ligand-free β2AR – β-arrestin-2 complex.

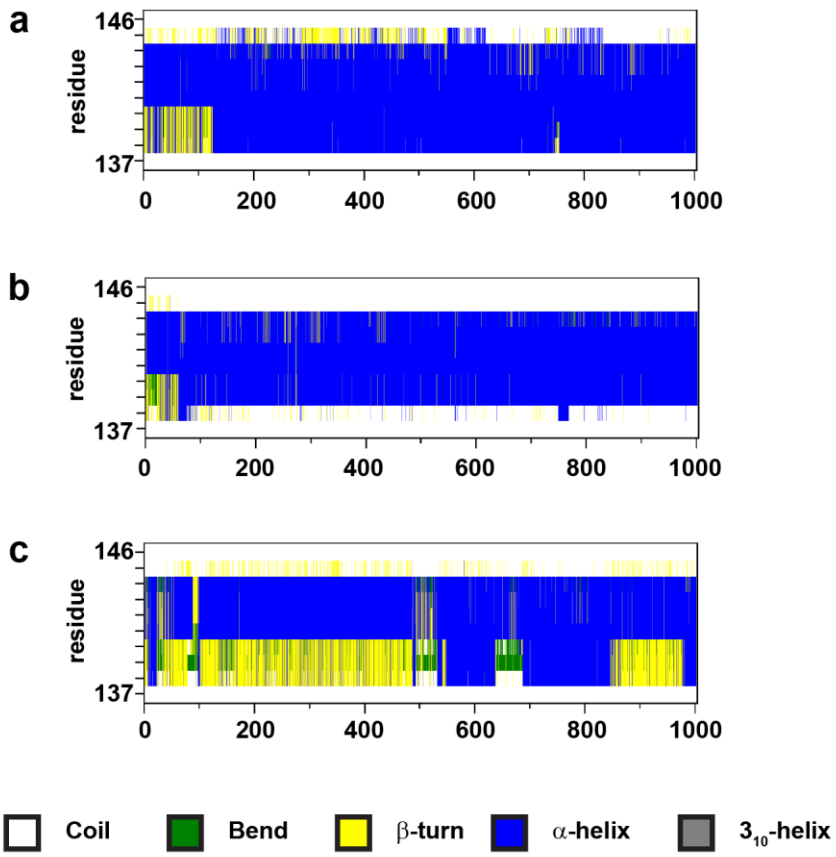

Figure S13. Evolution of the secondary structure of ICL2 during simulations. (**a**) active β2AR – Gs protein –epinephrine complex, 2nd replica; (**b**) active β2AR – Gs protein – epinephrine complex, 3rd replica; (**c**) active. β2AR – Gs protein – epinephrine complex, with restrained epinephrine and GDP.

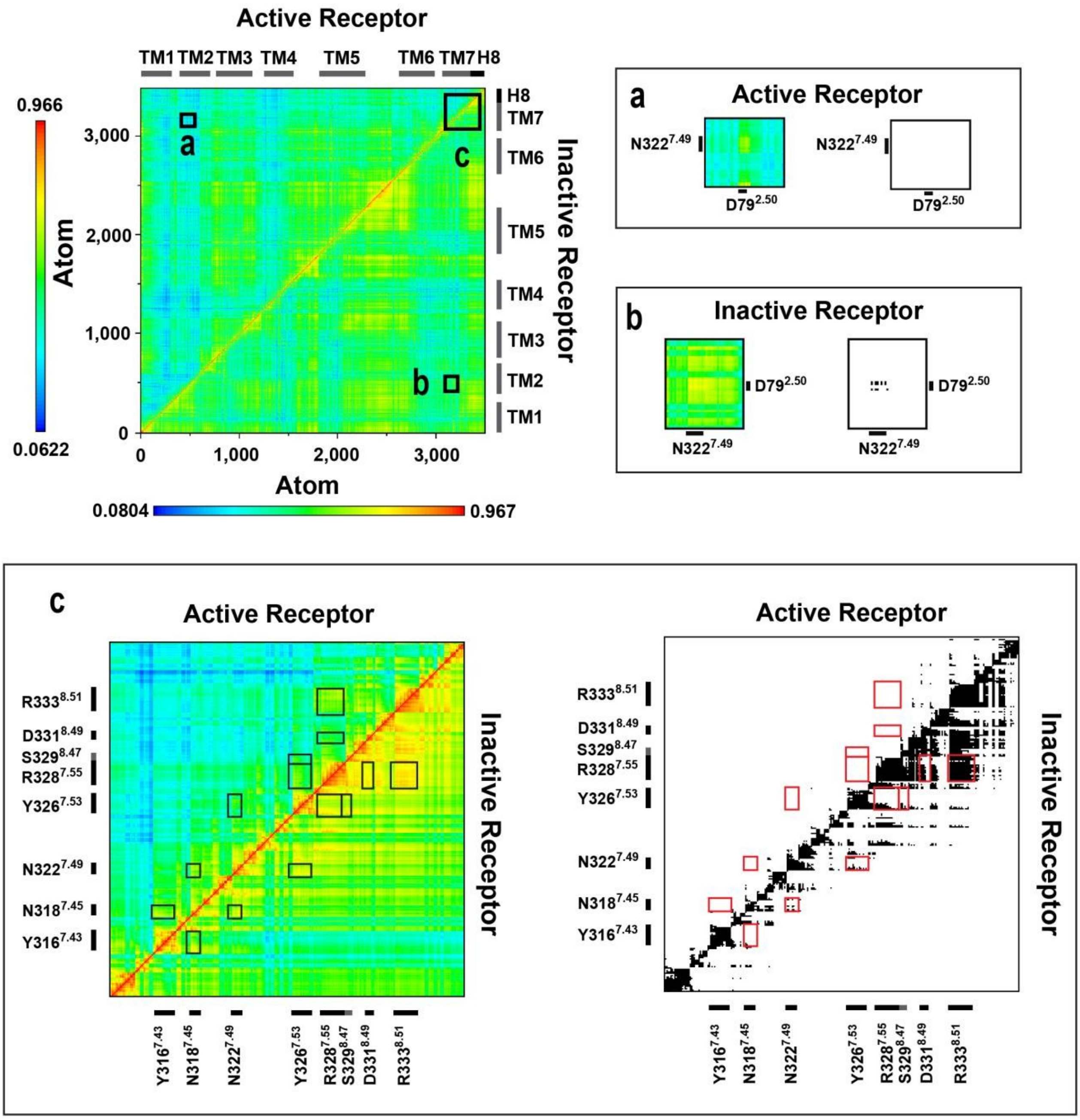

Figure S14. Representative dynamic cross-correlation matrices of the Gs protein-bound β2AR in the active and inactive, ligand-free states. Panels (**a**–**c**) are magnified views of regions of amino acid residues of interest. Black and white panels show correlations above the threshold of 0.65 MI. Dynamic cross-correlation matrices of all studied systems are available upon request.

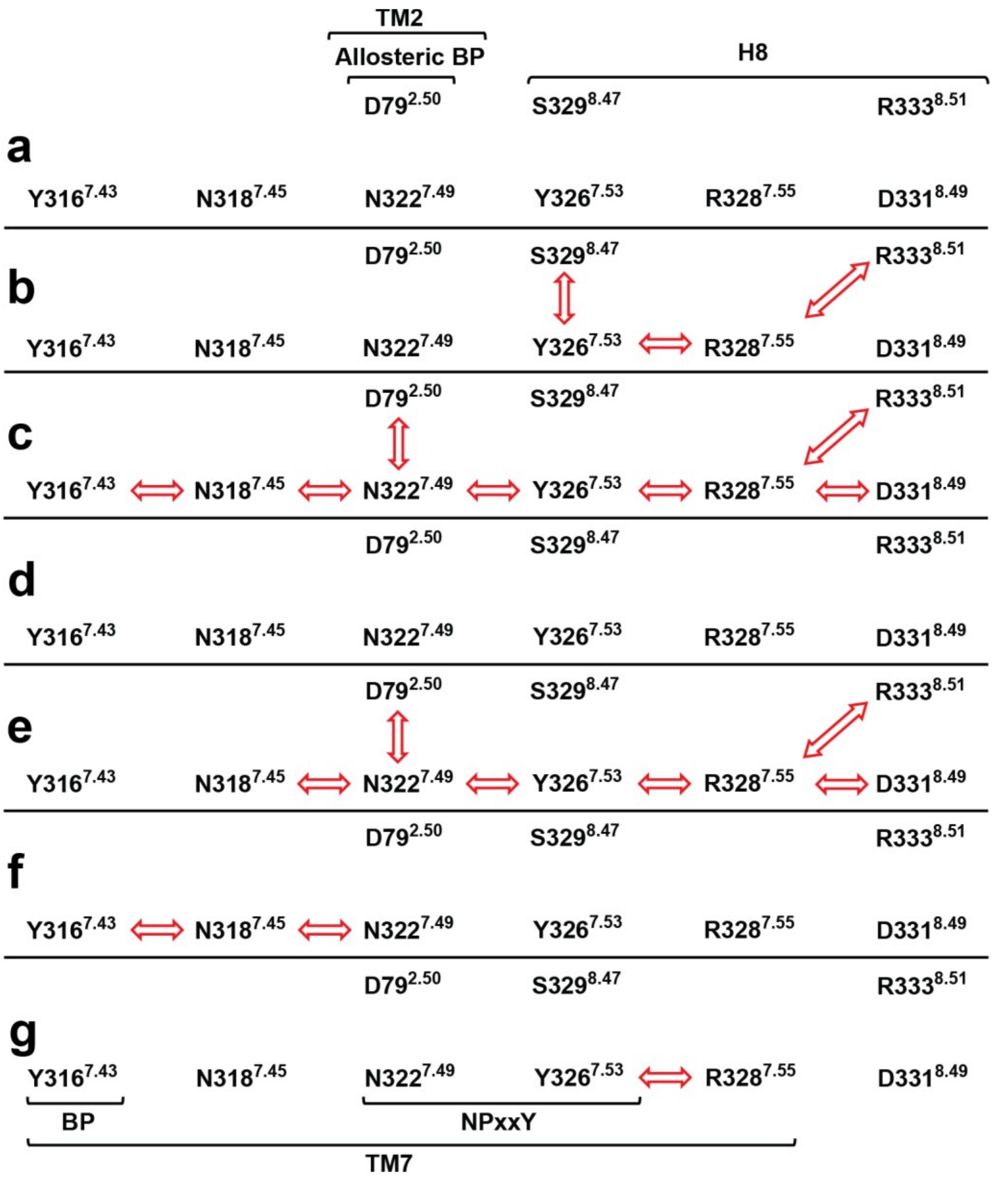

Figure S15. The polar signaling channel of the Gs protein or β-arrestin-2-bound β2AR analyzed by dynamic cross correlation analysis. (**a**) active β2AR – Gs protein – epinephrine complex, 2nd replica; (**b**) active β2AR – Gs protein – epinephrine complex, 3rd replica; (**c**) active β2AR – Gs protein – epinephrine complex, with restrained epinephrine and GDP; (**d**) active, ligand-free ◎2AR – Gs protein complex; (**e**) inactive, ligand-free. β2AR – Gs protein complex; (**f**) active, ligand-free β2AR – β-arrestin-2 complex; (**g**) inactive, ligand-free β2AR– β-arrestin-2 complex. Red arrows indicate correlated motions of the respective amino acids.
